# An optimal consumption and investment problem with quadratic utility and negative wealth constraints

**DOI:** 10.1186/s13660-017-1469-x

**Published:** 2017-08-15

**Authors:** Kum-Hwan Roh, Ji Yeoun Kim, Yong Hyun Shin

**Affiliations:** 1 Department of Mathematics, Hannam University, Daejeon, 34430 Republic of Korea; 2 Department of Mathematics, Sookmyung Women’s University, Seoul, 04310 Republic of Korea

**Keywords:** consumption, portfolio selection, quadratic utility, negative wealth constraints, martingale method

## Abstract

In this paper, we investigate the optimal consumption and portfolio selection problem with negative wealth constraints for an economic agent who has a quadratic utility function of consumption and receives a constant labor income. Due to the property of the quadratic utility function, we separate our problem into two cases and derive the closed-form solutions for each case. We also illustrate some numerical implications of the optimal consumption and portfolio.

## Introduction

We provide an optimal consumption and portfolio decision with negative wealth constraints for an economic agent who has a quadratic utility function of consumption. A bliss level of consumption is an import feature of the quadratic utility. It means that an agent’s risk taking becomes zero at a wealth level for some bliss point of consumption. When the wealth level exceeds a bliss point, her consumption does not increase. In this model, we derive an analytic solution with the negative wealth constraint. And we check some properties of optimal consumption and portfolio with a constant labor income.

After Merton’s seminal works [1, 2], many researchers have studied an optimal consumption and portfolio selection problem with various realistic constraints. Merton [1, 2] solved the portfolio optimization problem of an agent who has a Hyperbolic Absolute Risk Aversion (HARA) type utility function. However, he has not considered the labor income of an agent. Park and Jang [3] studied the optimal consumption, investment and retirement strategies with negative wealth constraints. But they considered the agent whose utility function is Constant Relative Risk Aversion (CRRA).

Koo et al. [4] and Shin et al. [5] considered an optimal consumption and portfolio selection problem of an agent who has a quadratic utility function and faces a subsistence consumption constraint. However, they did not consider the agent’s labor income and the negative wealth constraint. Here, negative wealth constraints contain the borrowing constraints which mean the restriction of a loan. Lim and Shin [6] and Shim [7] derived the closed-form solutions of optimal consumption and portfolio with general utility. Since the quadratic utility does not satisfy the strictly concave property, our results are different from theirs.

The rest of the paper is organized as follows. In Section 2, we illustrate the financial market setup. In Section 3, we obtain the optimal policies of our optimization problem with two cases ($\hat{y}>1$ or $\hat{y}<1$). Concluding remarks are given in Section 4.

## The financial market setup

We assume that there are two tradable assets in the financial market: one is a risk-free asset and the other is a risky asset. $r>0$ is a constant interest rate, and the price of the risky asset $S_{t}$ follows the geometric Brownian motion (GBM)

$$ dS_{t}=\mu S_{t}\, dt+\sigma S_{t}\, dB_{t}, $$

where $\mu>r$ and $\sigma>0$ are constants, $B_{t}$ is a standard Brownian motion on a complete probability space $(\Omega, \mathbb {P},\mathcal{F})$, and $\{\mathcal{F}_{t} \}_{t\geq0}$ is the $\mathbb{P}$-augmentation of the filtration generated by the standard Brownian motion $\{ B_{t} \}_{t\geq0}$.

Let $\pi_{t}$ be the amount invested in the risky asset at time *t* and $c_{t}$ be consumption at time *t*. A portfolio process $\{ \pi _{t} \}_{t\geq0}$ is a measurable and adapted process with respect to $\{\mathcal{F}_{t} \}_{t\geq0}$, and a consumption process $\{ c_{t} \}_{t\geq0}$ is a measurable and adapted nonnegative process with respect to $\{\mathcal{F}_{t} \} _{t\geq0}$. They satisfy the following mathematical conditions:

$$\int_{0}^{t} \pi_{s}^{2} \,ds< \infty\quad \mbox{and} \quad \int_{0}^{t} c_{s} \,ds< \infty\quad \mbox{for all } t\geq0 \text{ a.s.} $$

Let $X_{t}$ be the agent’s wealth process at time *t* that evolves according to the following stochastic differential equation (SDE):

2.1 $$ dX_{t}=\bigl[rX_{t}+\pi_{t}( \mu-r)-c_{t}+I\bigr]\,dt+\sigma\pi_{t} \,dB_{t}, $$

with an initial endowment $X_{0}=x$ and a constant labor income $I>0$.

From the wealth process (2.1), we can derive the following budget constraint:

2.2 $$ \mathbb{E} \biggl[ \int_{0}^{\infty}H_{t}(c_{t}-I)\,dt \biggr]\leq x, $$

where $\theta:=(\mu-r)/\sigma$ is the market price of risk, and $H_{t}:=e^{- (r+\frac{1}{2}\theta^{2} )t-\theta B_{t}}$ is the pricing kernel.

In this paper, we also consider the following negative wealth constraint (see [3]):

2.3 $$ X_{t}\geq-\nu\frac{I}{r} \quad \mbox{for all } t \geq0 \text{ and } \nu\in[0,1]. $$

This restriction means that the agent can borrow partially against her future labor income, that is, when $\nu=0$, she cannot borrow against her future labor income (this is called a nonnegative wealth constraint or borrowing constraint), and $\nu=1$ implies that she can borrow fully against her future labor income.

## The optimization problem

Now we consider our optimization problem. In this problem, the infinitely-lived agent wants to maximize her expected lifetime utility:

3.1 $$ V(x):=\sup_{(c,\pi)\in\mathcal{A}(x)} \mathbb{E} \biggl[ \int _{0}^{\infty}e^{-\rho t}\bigl(c_{t}-Rc^{2}_{t} \bigr)\,dt \biggr] $$

subject to the budget constraint (2.2) and the negative wealth constraint (2.3). Here, $\rho>0$ is a subjective discount factor, $R>0$ is the constant coefficient of the quadratic utility function, and $\mathcal{A}(x)$ is the class of all admissible controls $(c,\pi)$ at *x*.

### Remark 3.1

Due to the feature of the quadratic utility function, there is a bliss consumption level $\bar{c}:={1}/{(2R)}$, and the negative wealth constraint level should be lower than the bliss wealth level, that is,

3.2 $$ -\nu\frac{I}{r}< \frac{1}{2rR}-\frac{I}{r}. $$

This assumption is deduced from the bliss level which is one of the aspects of quadratic utility. The right-hand side of inequality (3.2) is bliss wealth level *x̄* which is obtained as follows:

3.3 $$ \bar{x}= \int_{0}^{\infty}e^{-rt}(\bar{c}-I)\,dt= \frac{1}{2rR}-\frac{I}{r}. $$

We denote by *ũ* the dual utility function of a quadratic utility function, which is defined by

$$\begin{aligned} \tilde{u}(y)&:=\sup_{c} \bigl\{ c-Rc^{2}-y(c-I) \bigr\} \\ &= \biggl\{ \frac{(1-y)^{2}}{4R} +yI \biggr\} \mathbf{1}_{\{0< y< 1\}}+yI \mathbf{1}_{\{y\geq1\}}, \end{aligned}$$

where $\mathbf{1}_{A}$ is an indicator function. For a domain $\{0< y<1\} $, the optimal consumption of the agent is $(1-y)/(2R)$ and, for a domain $\{y\geq1\}$, the agent consumes nothing, that is, the optimal consumption is zero.

For a Lagrange multiplier $\lambda>0$, we define a dual value function as follows:

$$\begin{aligned} \tilde{V}(\lambda)&=\sup_{c} \mathbb{E} \biggl[ \int_{0}^{\infty}e^{-\rho t}\bigl\{ c_{t}-Rc_{t}^{2}\bigr\} \,dt-\lambda \int_{0}^{\infty}H_{t}(c_{t}-I)\,dt \biggr] \\ &=\sup_{c} \mathbb{E} \biggl[ \int_{0}^{\infty}e^{-\rho t}\bigl\{ c_{t}-Rc_{t}^{2}-\lambda e^{\rho t} H_{t}(c_{t}-I)\bigr\} \,dt \biggr] \\ &=\mathbb{E} \biggl[ \int_{0}^{\infty}e^{-\rho t}\tilde{u}(y_{t}) \,dt \biggr], \end{aligned}$$

where $y_{t}:=\lambda e^{\rho t} H_{t}$ and the process $y_{t}$ follows the SDE

$$dy_{t}=(\rho-r)y_{t}\, dt-\theta y_{t}\, dB_{t}. $$

### Remark 3.2

For later use, we define the following quadratic equation:

3.4 $$ g(m):=\frac{1}{2}\theta^{2}m^{2}+ \biggl(\rho-r-\frac{1}{2}\theta ^{2} \biggr)m-\rho=0, $$

with two roots $m_{+}>1$ and $m_{-}<0$.

Now we consider the function

$$ \phi(t,y):=\mathbb{E} \biggl[ \int_{t} ^{\infty}e^{-\rho s} \biggl\{ \biggl( \frac{(1-y_{s})^{2}}{4R} +y_{s}I \biggr) \mathbf{1}_{\{0< y_{s}< 1\} }+y_{s}I \mathbf{1}_{\{y_{s}\geq1\}} \biggr\} \,ds\Big| y_{t}=y \biggr]. $$

By the Feymann-Kac formula, we derive the partial differential equations (PDEs) as follows:

$$\textstyle\begin{cases} \mathcal{L} \phi(t,y)+e^{-\rho t} [\frac{(1-y)^{2}}{4R} +yI ]=0 & \text{if }0< y< 1, \\ \mathcal{L} \phi(t,y)+e^{-\rho t}yI=0 & \text{if } y>1, \end{cases} $$

where the partial differential operator is given by

$$\mathcal{L}:=\frac{\partial}{\partial t}+(\rho-r)y\frac{\partial }{\partial y}+\frac{1}{2} \theta^{2} y^{2}\frac{\partial^{2}}{\partial y^{2}}. $$

If we conjecture that $\phi(t,y)=e^{-\rho t} v(y)$, then we derive the following ordinary differential equations (ODEs):

3.5 $$ \textstyle\begin{cases} \frac{1}{2}\theta^{2} y^{2} v''(y)+(\rho-r)y v'(y)-\rho v(y)+\frac {(1-y)^{2}}{4R} +yI=0 & \text{if } 0< y< 1, \\ \frac{1}{2}\theta^{2} y^{2} v''(y)+(\rho-r)y v'(y)-\rho v(y)+yI=0 & \text{if } y>1 . \end{cases} $$

We define $\hat{y}>0$ as the level of a dual variable of the negative wealth constraint level $-\nu{I}/{r}$.

### Remark 3.3

We define the positive constant *K* for indicating that *ŷ* is less or greater than 1.

3.6 $$ K:=\frac{1}{2R(m_{+}-1)}\frac{2-m_{+}}{\rho-2r+\theta^{2}}>0. $$

By comparison with the range from wealth constraint to the bliss level of wealth and *K*, we can check that $\hat{y}>1$ or $\hat{y}<1$. And we will solve ODEs (3.5) with two cases: one is $\hat {y}>1$, and the other is $\hat{y}<1$.

### In the case of $\hat{y}>1$

If $\hat{y}>1$, we can rewrite ODEs (3.5) as

3.7 $$ \textstyle\begin{cases} \frac{1}{2}\theta^{2} y^{2} v''(y)+(\rho-r)y v'(y)-\rho v(y)+\frac {(1-y)^{2}}{4R} +yI=0 & \text{if } 0< y< 1, \\ \frac{1}{2}\theta^{2} y^{2} v''(y)+(\rho-r)y v'(y)-\rho v(y)+yI=0 & \text{if } 1< y< \hat{y}. \end{cases} $$

#### Proposition 3.1

*The solutions to ODEs* (3.7) *are given by*

3.8 $$ v(y)= \textstyle\begin{cases} D_{1}y^{m_{+}}-\frac{1}{4R(\rho-2r+\theta^{2})}y^{2}+ (\frac {I}{r}-\frac{1}{2rR} )y+\frac{1}{4\rho R} &\textit {if } 0< y< 1 , \\ C_{1} y^{m_{+}}+C_{2}y^{m_{-}}+\frac{I}{r}y &\textit{if } 1< y< \hat{y} , \end{cases} $$

*where*

$$\begin{aligned}& C_{2} =\frac{1}{m_{+}-m_{-}} \biggl(-\frac{m_{+}-2}{4R(\rho-2r+\theta ^{2})}- \frac{m_{+}-1}{2r R}+\frac{m_{+}}{4\rho R} \biggr)>0, \\& \hat{y} = \biggl(-\frac{(1-\nu)(m_{+}-1)I}{C_{2} m_{-}(m_{+}-m_{-})r} \biggr)^{\frac{1}{m_{-} -1}}, \\& C_{1} =-\frac{C_{2} m_{-}(m_{-}-1)}{m_{+}(m_{+}-1)}\hat{y}^{m_{-}-m_{+}}< 0, \\& D_{1} =C_{1}+C_{2}+\frac{1}{4R(\rho-2r+\theta^{2})}+ \frac{1}{2r R}-\frac {1}{4\rho R}. \end{aligned}$$

#### Proof

From ODEs (3.7), we obtain the solution as follows:

$$ v(y)= \textstyle\begin{cases} D_{1}y^{m_{+}}+D_{2}y^{m_{-}}-\frac{y^{2}}{4R(\rho-2r+\theta^{2})}+ (\frac {I}{r}-\frac{1}{2rR} )y+\frac{1}{4\rho R} &\text{if } 0< y< 1 , \\ C_{1} y^{m_{+}}+C_{2}y^{m_{-}}+\frac{I}{r}y &\text{if } 1< y< \hat{y} , \end{cases} $$

where $m_{+}$ and $m_{-}$ are two roots of quadratic equation (3.4). Since, for $0< y<1$, the first equation has to satisfy the well-definedness, $D_{2}$ should be zero. By using the free boundary conditions of the negative wealth constraint, $v'(\hat{y})=\nu\frac {I}{r}$ and $v''(\hat{y})=0$, and $C^{1}$-condition of $v(y)$ at $y=1$, we derive the coefficients and *ŷ* as follows:

$$\begin{aligned}& C_{2} =\frac{1}{m_{+}-m_{-}} \biggl(-\frac{m_{+}-2}{4R(\rho-2r+\theta ^{2})}- \frac{m_{+}-1}{2r R}+\frac{m_{+}}{4\rho R} \biggr)>0, \\& \hat{y} = \biggl(-\frac{(1-\nu)(m_{+}-1)I}{C_{2} m_{-}(m_{+}-m_{-})r} \biggr)^{\frac{1}{m_{-} -1}}, \\& C_{1} =-\frac{C_{2} m_{-}(m_{-}-1)}{m_{+}(m_{+}-1)}\hat{y}^{m_{-}-m_{+}}< 0, \\& D_{1} =C_{1}+C_{2}+\frac{1}{4R(\rho-2r+\theta^{2})}+ \frac{1}{2r R}-\frac {1}{4\rho R} \end{aligned}$$

(we will show that $C_{2}>0$ in Proposition 3.3). □

By the Legendre inverse transform formula, the value function $V(\cdot )$ can be obtained as follows:

3.9 $$ V(x)=\min_{\lambda>0} \bigl\{ v(\lambda)+\lambda x \bigr\} . $$

#### Theorem 3.1

*The value function* $V(x)$ *of optimization problem* (3.1) *is given by*

$$ V(x)= \textstyle\begin{cases} (1-m_{+})C_{1} \zeta^{m_{+}}+(1-m_{-})C_{2}\zeta^{m_{-}} &\textit{if } -\nu\frac {I}{r} \leq x< \tilde{x}, \\ (1-m_{+})D_{1}\xi^{m_{+}}+\frac{\xi^{2}}{4R(\rho-2r+\theta^{2})}+\frac {1}{4\rho R} &\textit{if } \tilde{x} \leq x< \bar{x} , \\ \frac{1}{4\rho R} & \textit{if } x\geq\bar{x} , \end{cases} $$

*where*

$$\begin{aligned}& \tilde{x}=-C_{1}m_{+}-C_{2}m_{-} -\frac{I}{r}, \\& \bar{x}=\frac{1}{2rR}-\frac{I}{r}, \end{aligned}$$

*ξ* *and* *ζ* *are the solutions to the algebraic equations*

$$\begin{aligned}& x=-C_{1} m_{+}\zeta^{m_{+} -1}-C_{2} m_{-} \zeta^{m_{-} -1} -\frac{I}{r}, \\& x=-D_{1} m_{+} \xi^{m_{+}-1}+\frac{1}{2R(\rho-2r+\theta^{2})}\xi+ \frac {1}{2rR}-\frac{I}{r}, \end{aligned}$$

*respectively*.

#### Proof

The first order condition for equation (3.9) takes the following form:

$$x=-v'(\lambda), $$

where the function $v(\cdot)$ is described in Proposition 3.1. Substituting the first order condition into equation (3.9), we can complete the proof. □

When $\hat{y}>1$, we can provide the optimal strategy $(c,\pi)$.

#### Theorem 3.2

*The optimal strategies are given by*

$$ c^{*}= \textstyle\begin{cases} 0 &\textit{if } -\nu\frac{I}{r} \leq x< \tilde{x} , \\ \frac{1-\xi}{2R} &\textit{if } \tilde{x} \leq x< \bar{x} , \\ \frac{1}{2R}& \textit{if } x\geq\bar{x} , \end{cases} $$

*and*

$$ \pi^{*}= \textstyle\begin{cases} \frac{\theta}{\sigma} (C_{1}m_{+}(m_{+} -1)\zeta^{m_{+} -1}+C_{2}m_{-}(m_{-} -1)\zeta^{m_{-} -1} ) &\textit{if } -\nu\frac{I}{r} \leq x< \tilde {x} , \\ \frac{\theta}{\sigma} (D_{1}m_{+}(m_{+} -1)\xi^{m_{+} -1}-\frac {1}{2R(\rho-2r+\theta^{2})} \xi ) &\textit{if } \tilde{x} \leq x< \bar{x} , \\ 0& \textit{if } x\geq\bar{x} . \end{cases} $$

#### Proof

By the duality of value function and the Itô formula, we can obtain the following equation:

3.10 $$ dX_{t}= \biggl(-v''(y) ( \rho-r)y-\frac{1}{2}v'''(y) \theta^{2} y^{2} \biggr)\,dt+\theta y v''(y) \,dB_{t}. $$

By comparing (2.1) and (3.10), we derive the optimal consumption $c_{t}^{*}$ and portfolio $\pi_{t}^{*}$ as follows:

3.11 $$ \begin{aligned} &c_{t}^{*}=-rv'(y)+ \bigl(\rho-r+\theta^{2}\bigr)yv''(y)+ \frac{1}{2}\theta ^{2}y^{2}v'''(y)+I, \\ &\pi_{t}^{*}=\frac{\theta}{\sigma}yv''(y). \end{aligned} $$

By substituting equation (3.8) into (3.11), we can derive the optimal consumption and portfolio. □

#### Proposition 3.2

*If the following inequality* (3.12) *holds*,

3.12 $$ \frac{1}{2rR}-\frac{I}{r}- \biggl(-\nu \frac{I}{r} \biggr)>\frac {1}{2R(m_{+}-1)}\frac{2-m_{+}}{\rho-2r+\theta^{2}}=:K, $$

*where* *K* *is given in* (3.6), *then*

$$\hat{y}= \biggl(-\frac{(1-\nu)(m_{+}-1)I}{C_{2} m_{-}(m_{+}-m_{-})r} \biggr)^{\frac{1}{m_{-} -1}}>1. $$

#### Proof

Since $m_{-}<0$, it is enough to show that

$${-\frac{C_{2} m_{-}(m_{+}-m_{-})r}{(1-\nu)(m_{+}-1)I}>1}. $$

This inequality implies

3.13 $$ \frac{1}{2rR}-\frac{I}{r}- \biggl(-\nu \frac{I}{r} \biggr)>\frac {1}{2rR} \biggl(\frac{r}{2} \frac{m_{-}(2-m_{+})}{(m_{+}-1)(\rho-2r+\theta ^{2})}+(1-m_{-})+\frac{rm_{+} m_{-}}{2\rho(m_{+}-1)} \biggr). $$

From the difference of the right-hand side of inequalities (3.12) and (3.13), we have

3.14 $$ \frac{1}{2rR} \biggl(\frac{r(2-m_{+})(m_{-}-2)}{2(m_{+}-1)(\rho-2r+\theta ^{2})}+(1-m_{-})+ \frac{rm_{+} m_{-}}{2\rho(m_{+}-1)} \biggr). $$

By the relation between roots and coefficients of quadratic equation (3.4), we can check that equation (3.14) should be zero, that is, $\hat{y}>1$. □

#### Proposition 3.3

*In Proposition* 3.1, $C_{2}\geq0$.

#### Proof

Refer to Koo et al. [4]. □

### In the case of $\hat{y}<1$

If $\hat{y}<1$, *y* does not exceed *ŷ*. So we obtain the following ODE from ODEs (3.5).

3.15 $$ \frac{1}{2}\theta^{2} y^{2} v''(y)+(\rho-r)y v'(y)-\rho v(y)+ \frac {(1-y)^{2}}{4R} +yI=0 \quad \text{if } 0< y< \hat{y}. $$

#### Proposition 3.4

*The solution to ODE* (3.15) *is given by*

3.16 $$ v(y)=D_{1}y^{m_{+}}-\frac{y^{2}}{4R(\rho-2r+\theta^{2})}+ \biggl( \frac {I}{r}-\frac{1}{2rR} \biggr)y+\frac{1}{4\rho R} \quad \textit{if } 0< y< \hat{y}, $$

*where*

$$\begin{aligned}& \hat{y} =2R\bigl(\rho-2r+\theta^{2}\bigr) \biggl(\frac{m_{+}-1}{m_{+}-2} \biggr) \biggl((1-\nu)\frac{I}{r}-\frac{1}{2rR} \biggr), \\& D_{1} =\frac{1}{2R(\rho-2r+\theta^{2})(m_{+}-1)m_{+}}\hat{y}^{2-m_{+}}. \end{aligned}$$

#### Proof

For $0< y<\hat{y}$, similar to the proof of Proposition 3.1, we derive the solution $v(\cdot)$ in (3.16). By the free boundary conditions at $y=\hat{y}$, we obtain two equations $v'(\hat {y})=\nu{I}/{r}$ and $v''(\hat{y})=0$. Using these equations, we have

$$\begin{aligned}& \hat{y} =2R\bigl(\rho-2r+\theta^{2}\bigr) \biggl(\frac{m_{+}-1}{m_{+}-2} \biggr) \biggl((1-\nu)\frac{I}{r}-\frac{1}{2rR} \biggr), \\& D_{1} =\frac{1}{2R(\rho-2r+\theta^{2})(m_{+}-1)m_{+}}\hat{y}^{2-m_{+}}. \end{aligned}$$

 □

By the duality of value function $V(x)$, we derive the value function $V(\cdot)$.

#### Theorem 3.3

*When* $\hat{y}<1$, *the value function* $V(x)$ *of optimization problem* (3.1) *is given by*

$$ V(x)= \textstyle\begin{cases} (1-m_{+})D_{1}\xi^{m_{+}}+\frac{\xi^{2}}{4R(\rho-2r+\theta^{2})}+\frac {1}{4\rho R} &\textit{if } -\nu\frac{I}{r} \leq x< \bar{x} , \\ \frac{1}{4\rho R} & \textit{if } x\geq\bar{x} , \end{cases} $$

*where*

$$\bar{x}=\frac{1}{2rR}-\frac{I}{r}, $$

*and* *ξ* *is the solution to the algebraic equation*

$$ x=-D_{1} m_{+} \xi^{m_{+}-1}+\frac{1}{2R(\rho-2r+\theta^{2})}\xi+ \frac {1}{2rR}-\frac{I}{r}. $$

#### Theorem 3.4

*The optimal strategies are given by*

$$ c^{*}= \textstyle\begin{cases} \frac{1-\xi}{2R} &\textit{if } -\nu\frac{I}{r} \leq x< \bar{x} , \\ \frac{1}{2R}& \textit{if } x\geq\bar{x}, \end{cases} $$

*and*

$$ \pi^{*}= \textstyle\begin{cases} \frac{\theta}{\sigma} (D_{1}m_{+}(m_{+} -1)\xi^{m_{+} -1}-\frac {1}{2R(\rho-2r+\theta^{2})} \xi ) &\textit{if } -\nu\frac{I}{r} \leq x< \bar{x} , \\ 0& \textit{if } x\geq\bar{x} . \end{cases} $$

#### Proposition 3.5

*If the following inequality holds*, *then* $\hat{y}<1$.

3.17 $$ \frac{1}{2rR}-\frac{I}{r}- \biggl(-\nu \frac{I}{r} \biggr)< \frac {1}{2R(m_{+}-1)}\frac{2-m_{+}}{\rho-2r+\theta^{2}}=:K, $$

*where* *K* *is given in* (3.6).

#### Proof

Since the signs of $\rho-2r+\theta^{2}$ and $2-m_{+}$ are the same, equation (3.17) is rewritten as follows:

$$\hat{y}=2R\bigl(\rho-2r+\theta^{2}\bigr)\frac{m_{+}-1}{2-m_{+}} \biggl( \frac {1}{2rR}-\frac{I}{r}- \biggl(-\nu\frac{I}{r} \biggr) \biggr)< 1. $$

 □

#### Remark 3.4

**Figure 1:**
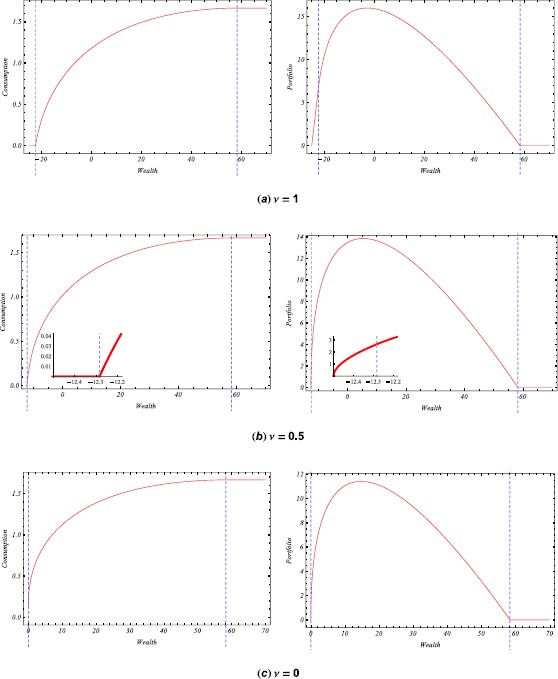
**The optimal consumption and portfolio as a function of wealth (** $\pmb{\rho=0.04}$ **,** $\pmb{r=0.02}$ **,** $\pmb{\mu=0.05}$ **,** $\pmb{\sigma=0.2}$ **,** $\pmb{I=0.5}$ **,** $\pmb{R=0.3}$ **).**

In Figure 1, we plot the optimal consumption and portfolio with respect to the wealth (especially, small figures in Figure 1(b) represent the specific region of wealth level from −12.5 to −12.2). From the figures, we can see the effects of the bliss wealth level and the threshold *ŷ* ($\nu=1, 0.5$: $\hat{y}>1$ and $\nu=0$: $\hat{y}<1$). The left dotted line of each figure represents the wealth level at which optimal consumption is zero, and the right dotted line represents the bliss wealth level.

Basically, we obtain similar results to those of Koo et al. [4], that is, if $\hat{y}>1$, then the optimal consumption is zero until wealth reaches the threshold wealth level corresponding to the dual variable $y=1$. After wealth reaches this level, the optimal consumption increases as wealth increases. But after the bliss level *x̄*, the optimal consumption stays at $\bar{c}=1/(2R)$. For the optimal portfolio, it increases from zero to the certain maximum until wealth reaches a certain wealth level. The optimal portfolio decreases above this level and becomes zero above the bliss level *x̄*.

If $0<\hat{y}<1$, however, there is no zero consumption region. So the optimal consumption increases as wealth increases. But after the bliss level *x̄*, the optimal consumption stays at $\bar{c}=1/(2R)$. For the optimal portfolio, we see behavior similar to the case of $\hat{y}>1$.

## Concluding remarks

We solve the optimal consumption and investment problem with negative wealth constraints. Negative wealth constraints are the general borrowing constraints against future labor income. We consider the optimization problem when an agent receives a constant labor income and has quadratic utility. We use the martingale duality approach to obtain the closed-form solutions and illustrate the effects of the proportion *ν* of the wealth constraint on the optimal consumption and portfolio.
